# Financial inclusion for people with disability: a scoping review

**DOI:** 10.1080/16549716.2024.2342634

**Published:** 2024-05-10

**Authors:** Louise Puli, Natasha Layton, Diane Bell, Abu Zafar Shahriar

**Affiliations:** a Monash Rehabilitation, Ageing and Independent Living Research Centre, Monash University, Melbourne, Australia; b Business School, Stellenbosch University, Stellenbosch, South Africa; c Computer Science, University College London, London, UK; d Department of Banking and Finance, Monash University, Melbourne, Australia

**Keywords:** Financial inclusion, ICT, disability, banking, assistive technology, training, exclusion

## Abstract

**Background:**

Financial exclusion is a human rights issue affecting health equity. Evidence demonstrates that financial exclusion is exacerbated for people with disability and those in low- to middle-income countries (LMIC). Barriers to financial access include limited demand for services, banking inadequacies in catering to people with disability, and insufficiently accessible information technologies (ICT) and infrastructure.

**Objectives:**

This scoping review sought to identify barriers to and facilitators of financial inclusion for people with disability in LMIC. As a secondary objective, the study explored the potential of financial education and ICT utilisation as viable strategies for enhancing financial inclusion.

**Methods:**

This review utilised the Arksey and O’Malley framework and PRISMA Checklist for systematic literature examination and data extraction. The WHO’s Environmental Factors guided the analysis to propose potential interventions and to generate recommendations.

**Results:**

The review analysed 26 publications from various global regions and fields including finance, business, technology, health and disability policy. It identified consistent financial inclusion barriers for people with disability, resulting in a set of global recommendations across attitudes, environment, technology, services, and policy.

**Conclusions:**

Recommendations include using ICT, digital innovation and multi-stakeholder collaboration to address the financial barriers experienced by people with disability. These efforts, rooted in social justice, aim to include people with disability in LMIC as valued financial sector participants, promoting health and equity.

## Paper context

- **Main findings:** There are global access barriers and enablers to financial inclusion for people living with disability. Recommendations to improve access include countering stigma and attitudinal barriers, engaging in user centred design of financial services,providing financial education and ensuring accessibility of assistive technology and ICT, along with the physical environment of the bank.
- **Added knowledge:** This study reviews the literature and offers a global overview of financial inclusion for people with disabilities, along with recommendations for universally applicable actions to enhance access.
- **Global health impact for policy and action:** Identifying barriers to financial inclusion and suggesting strategies to overcome them provides valuable guidance for policymakers and advocates working to improve access to financial services for people with disability.

## Background

Globally, 1.4 billion adults do not have an account with a formal financial institution, such as a bank, microfinance institution, post office, or mobile financial service provider, and are thus financially excluded [1]. Although national data on financial inclusion disaggregated by disability remain scarce, available data show that the proportion of people with disability who are not engaging in the financial sector, and classified as ‘unbanked’ is significantly higher than that of people without disability [1].

Financial inclusion is considered a top priority of many policymakers and development practitioners for its potential to contribute to poverty alleviation and inclusive economic growth [2]. Financial inclusion also features in at least five of the 17 Sustainable Development Goals (SDGs) set by the United Nations for 2030 [3], namely, poverty alleviation (Goal 1), achieving food security (Goal 2), reducing gender-based inequalities (Goal 5), achieving inclusive and sustainable economic growth (Goal 8), and building resilient infrastructure which leaves no one behind (Goal 9). People with disability face multiple barriers to participation, including financial participation [4], which is a major human rights issue as it limits equitable outcomes [5]. Health and wellbeing are linked to access to participation in life areas [6]. With appropriate accommodations, people with disability can exercise their legal rights to participate in daily activities, being afforded the opportunity to reach their full potential. Accommodations refer to adjustments to minimise the gap between a person’s capability and the demands of the environment [7].

To be financially included, people with disability require access to the financial products and services that meet their needs. These financial services typically include banking, credit, insurance, and financial advisory services [8] and should be readily available and provided in a responsible and sustainable way. This can be achieved by addressing the challenges (or difficulties) that are faced.

The World Health Organisation (WHO) categorises the barriers to financial inclusion faced by people with disability into five groups [9]: (1) People with disability have a lower demand for formal financial services than those without disability; (2) customers with disability are not expected and welcomed by banks; (3) more appropriate technologies are needed to overcome the communication barriers experienced by some people with disability as the current state of information and communication technology (ICT) fails to meet the web content accessibility requirements; (4) financial services are not tailored to meet the needs of people with disability, and (5) more accessible public infrastructure is needed to accommodate the inclusion of people with disability in the formal financial system.

Clearly, approaches to enhancing financial inclusion for people with disability must consider the multidimensional aspects of financial exclusion. A robust and relevant socio-environmental framework is required to capture the multiple groups of barriers identified above and to analyse potential intervention approaches. In this study, we adopt the WHO’s International Classification of Functioning, Disability, and Health (ICF) [6]. The ICF is an internationally adopted framework used to describe human functioning in the context of the environment. It is suitable for understanding and synthesising complex biopsychosocial and socio-ecological elements [10], providing a lens through which to categorise the data, as outlined in the methods section.

## Study purpose

The primary objective of this study was to identify the existing barriers to and facilitators of financial inclusion for people with disability in low- to middle-income countries (LMICs). As a secondary objective, the study explored the potential of financial education and Information and Communication Technology (ICT) utilisation as viable strategies for enhancing financial inclusion within this demographic.

## Methods

### Author positionality

The authors positionality includes experiences living and working in low- and middle-income countries, and backgrounds including health practitionership, health services, inclusive education, business and finance. None of the authors identify as person with lived experience of disability and acknowledge this limitation.

#### Approach to review

Following the framework originally outlined by Arksey and O’Malley [11], a five-stage scoping review was conducted: (1) identifying the research question, (2) identifying relevant studies, (3) selecting studies, (4) charting the data, and (5) collating, summarising and reporting the results. The outcomes are presented following the Preferred Reporting Items for Systematic Reviews and Meta-Analyses extension for Scoping Reviews (PRISMA) Checklist [12,13]. One of three inclusion criteria had to be met for articles to be included in this review: i) speaks to financial inclusion, ii) concerns disability and banking, and iii) involves inclusive banking interventions. Exclusion criteria included a lack of focus on finance at a personal level or where the background to the issues was presented without any substantive research or review content. The population of interest were people with disability.

#### Search strategy

A broad systematic search strategy was conducted with support from health and business librarians. Search terms were identified through consultation with experts to obtain a ‘gold set’ and then further refined following trial searches, resulting in the following search string: ((‘disabled persons’[MeSH] OR disabili* OR disabled OR impair*) AND (bank* OR ‘financial inclusion’ OR money) AND (ICT OR technology OR ‘phone bank*’ OR ‘mobile bank*’) AND (barrier* OR inclusi*)). Four academic databases identified as primary sources were included: OVID Medline, Web of Science, SCOPUS, and Business Source Direct, searched during March 2023. A search of policy documents, guidelines, and white papers was also undertaken via the Overton database. Each database was searched by using MeSH headings (if applicable), synonyms, wildcards, and truncations where appropriate. Reference lists from eligible papers were screened to uncover further relevant studies through forward and backward citation tracking.

#### Study screening and selection

**Figure 1.**
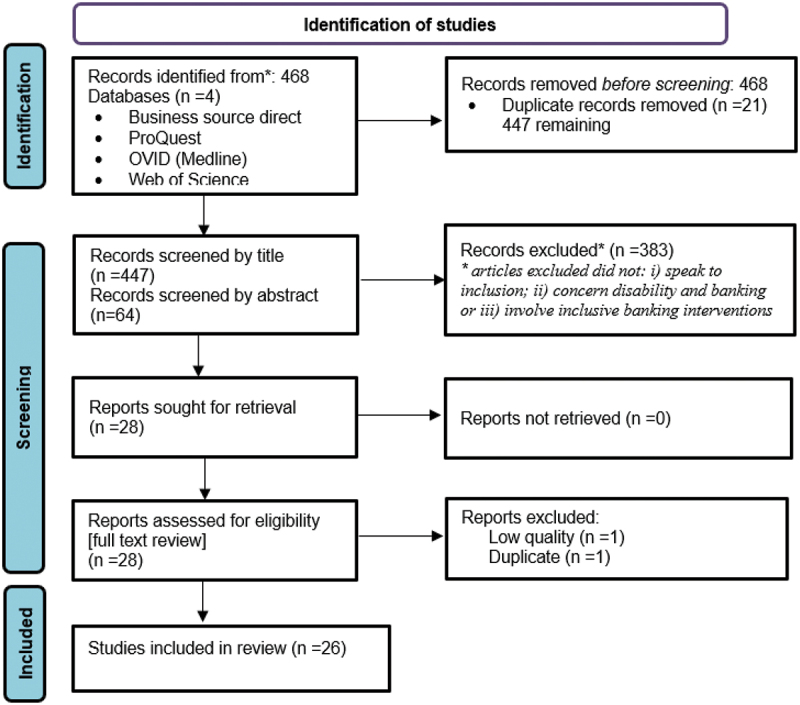
PRISMA diagram of scoping review yield.

The search yielded 468 records. Title and abstract screening by two independent reviewers resulted in 26 publications for full review and data extraction (Figure 1).

Results were compared, and disagreements were resolved through discussion and reaching consensus. Following the establishment of the final list of included articles, full-text screening was completed, with results entered into an Excel spreadsheet.

## Data charting

Extraction criteria were established in accordance with the two research objectives. These included document attributes such as title, authors, publication year, and source, as well as critical study details like geographical location, types of impairment under consideration (sensory, physical, cognitive), and intersectionality markers, such as poverty, the status of being in a lower-middle-income country, and access to universal health coverage.

## Data analysis

**Figure 2.**
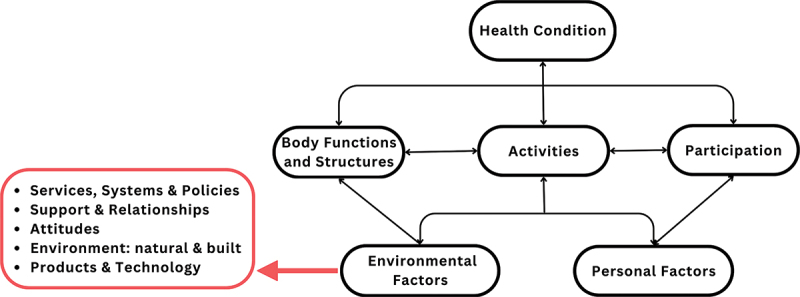
WHO ICF environmental factors adapted to form a coding framework.

The WHO’s ICF [14] environmental factor chapters comprise (1) attitudes, (2) the natural and built environment, (3) products and technology, (4) services, systems, and policies, and (5) support and relationships. These five chapters were adapted as a deductive framework to organise and analyse the data (Figure 2). Two researchers independently coded the data into barriers and facilitators against each chapter.

A meta-analysis of the studies was not possible due to the heterogeneity of scenarios, methods, and outcomes. Consistent with scoping review methods, risk of bias evaluations or critical appraisals of individual studies were not conducted. Rather, a narrative is provided that encapsulates common patterns and themes.

## Results

### Description of the included publications

The scoping review yielded 468 records, and after full screening, 26 publications (see Table 1) were included in the review. Published over two decades, from 2003 to 2023, most of the studies were journal articles, complemented by conference proceedings, case studies, research reports, and commentaries. Publications originated from diverse research fields, including risk and financial management, global business and technology, disability policy, and computer education. The reviewed materials encompass a broad geographical scope, representing various global regions such as the Western Pacific (e.g. Fiji), Southeast Asia (e.g. India, Bangladesh), Africa (e.g. Nigeria, Malawi), the Americas (e.g. USA, Canada), and Europe (e.g. Spain). Overall, studies included a variety of approaches to researching financial inclusion, and methodologies included reviews, using both quantitative and/or qualitative approaches. Many studies focussed on one type of impairment, for example, visual impairment, with five of the studies addressing the presence of a disability more generally across impairment types.

**Table 1.** Extracted articles.

No.	Reference	Document Type	Study design	Location	Type of impairment*	No. participants	Gender representation considered	Poverty considered
1	Acosta-Vargas et al.^31^	JA	RQN	Ecuador	PWD	Not stated	N	N
2	Alwi et al.^33^	JA	R	Global	NS	200	Y	N
3	Anderson et al.^34^	JA	RQL	Malawi	PWD	1,232	Y	Y
4	Asongu et al.^53^	JA	RQN	Africa (42 countries)	PWD	n/a	Y	Y
5	Barcellos et al.^36^	JA	RQN	United States of America	PWD	1,576	Y	Y
6	Bidarra and Seiji Oyamada^16^	JA	RQN	South Africa	V, Age	n/a	N	N
7	Martinson and Martinson^14^	JA	RQL	South Africa	V, H, M	90	N	Y
7	Chipere^54^	JA	MM	Developing countries	V	1	N	Y
8	Coduti et al.^24^	JA	RQL	United States of America	V, PWD	Not stated	N	N
9	Davies et al.^55^	JA	RQN	United States of America	COG	9	Y	N
10	Fuglerud and Dale^28^	JA	RQN	Norway	PWD, Age	Not stated	N	N
11	Gooding et al.^30^	JA	C	Nigeria	COG and M	n/a	N	N
12	Goundar and Sathye^21^	JA	MM	Fiji	V	21	N	N
13	Gyasi and Adam^56^	JA	MM	Ghana	Age	1,200	Y	N
14	Hedman et al.^57^	C	RQL	Sweden	Age, COG	37	Y	N
15	Jiya et al.^15^	JA	RQL	Malawi	PWD	10	Y	Y
16	Kaur and Dani^22^	JA	RQN	India	PWD	n/a	N	N
17	Pous et al.^58^	JA	MM	Spain	PWD	7	N	N
18	Mathew Martin and Rabindranath^51^	JA	RQN	India	PWD	103 websites	n/a	n/a
19	Okonji and Ogwezzy^23^	JA	RQL	Nigeria	V, Age	20	Y	Y
20	Polu et al.^35^	JA	RQL	Bangladesh	PWD	Not stated	Y	Y
21	Ripat et al.^27^	R	MM	Canada	PWD	24	N	N
22	Scott et al.^52^	JA	RQL	United States of America	COG	3	Y	N
23	Subekti et al.^29^	JA	MM	Indonesia	PWD, V	45	N	N
24	Wann and Burke-Smalley^18^	JA	RQL	United States of America	PWD	98,832	Y	Y
25	Xu^25^	JA	RQN	United States of America	PWD	23,861 households	N	Y
26	Yang and Lin^26^	JA	RQN	China	V	352	Y	Y

*included (sensory [vision/hearing]; physical [motor]; cognitive.

Abbreviations: Y= YES, N= NO; Type of publication: JA = Journal Article; F=Feature; B= Book (chapter);RQN= Research paper, quantitative RQL=Research paper, qualitative; MM= Mixed methods; R= Report; Design: S= Study; C= Commentary; R= Review; AT groups discussed: M= Mobility; SC= Self-Care; V= Vision; H= Hearing; COM= Communication; COG= Cognition; NS=Not stated.

### WHO ICF framework – data analysis

**Figure 3.**
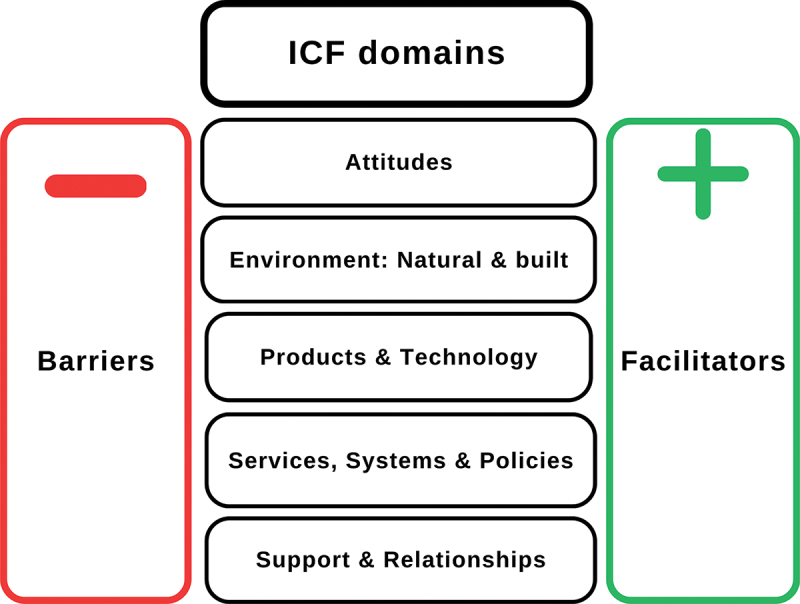
WHO ICF coding chapters: barriers and facilitators.

**Figure 4.**
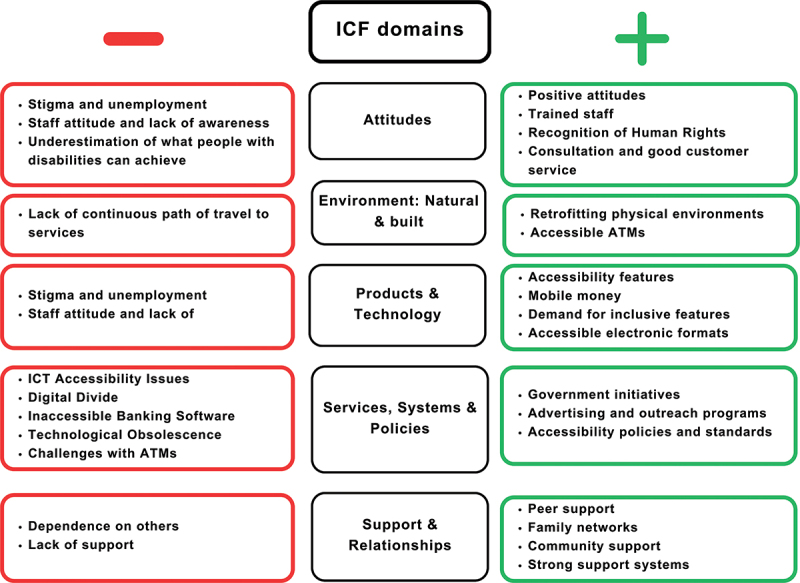
Examples of barriers and facilitators coded to 5 environmental factor chapters.

Data were coded using the five ICF environmental factors chapters: attitudes, the natural and built environment, products, and technology, services, systems, policies, support, and relationships (see Figure 3). Data for each theme are summarised below, with examples of barriers of facilitators depicted in Figure 4.

#### Attitudes

Both positive and negative attitudes play a crucial role in the financial inclusion of people with disability [14]. Negative societal attitudes and the stigma associated with disability, coupled with high unemployment rates, make banks less likely to view people with disability as valuable clientele, hence less likely to invest in accessibility mechanisms [14,15]. Often, financial institutions have few customers with disabilities, perpetuating the belief that there is no necessity to develop financial services specifically tailored to their needs [15,16]. Furthermore, due to high unemployment rates among people with disability, banks, insurance providers, and other financial institutions do not consider them valuable clients [16,17].

Given the lack of perceived value of people with disability as consumers of financial services, literature reports a lack of trained personnel globally who are equipped to communicate effectively with people with disability, in particular those with hearing or visual disabilities [16,18]. This extends not only to face-to-face interactions but also to written communication, telephone banking, and online services, which may not be accessible [16,19,20]. Low awareness and a lack of staff training results in some staff demonstrating impatience, an unwillingness to assist, or a lack of basic skills in interacting with people with disability, making banking ‘exceptionally stressful and frustrating’ [14,16]. The literature also strongly advocates for more robust consultation with people with disability when planning and designing financial services to harness facilitators and overcome barriers.

Encouraging examples are, however, also reported, with one such example being from Fiji, where positive attitudes towards people with disability are instrumental in promoting their financial inclusion; being a role model for other nations [21]. The Government of Fiji has recognised the importance of ensuring equal access to financial services for this population, demonstrating a commitment to inclusivity emphasised by prioritising financial inclusion as a crucial aspect of their policies.

#### Natural and built environment

The natural and built environment is frequently reported as a barrier to the financial inclusion of people with disability. Difficult terrains and inaccessible bank branches and automated teller machines (ATMs) pose challenges to accessing financial services [15], which can be mitigated or overcome by retrofitting physical environments and providing accessible ATMs [22]. Modifying infrastructures to accommodate the needs of people with disability can significantly improve their access to financial services. This includes implementing accessible pathways, tactile signage, and properly designed counters, ensuring a more inclusive environment for all customers [14,23]. These measures enable people with disability to navigate the environment and engage in financial transactions independently, promoting greater inclusivity. Ideally, however, inclusive design (including inclusive infrastructure) should be considered and incorporated at the outset, rather than retrofitting.

#### Products and technology

Products and technology can also enable or prohibit financial inclusion. The ‘digital divide’ refers to the fact that many people with disability may not possess a mobile phone (basic or smart) or have access to internet connectivity [24]. Lack of access leads to unfamiliarity with digital technology, which further restricts access to online banking services [25,26]. This digital divide is further compounded by the rapid pace of technological advancement [26,27]. The constant evolution of products, often without consultation with nor advising consumers, can leave previous access methods unusable [28]. As learning to navigate new technologies takes more time for those with disabilities, this continuous turnover further exacerbates the problem.

It is thus crucial to not only incorporate inclusive (human-centred) design principles when developing technology for accessing financial services, ensuring they are user-friendly and accessible to people with disability over time, but to do this through engagement with the assistive technology users and/or Organisations for Persons with Disabilities (OPDs). This not only includes the initial learning and adaptation period but also future adaptations required as technology evolves [14,15].

The use of ICT for security, e.g. to log in, is demonstrated to cause significant issues for individuals with visual or motor impairments who may be using assistive technology to access ICT-based systems. The use of audio or visual authentication, e.g. CAPTCHAs, may render official banking websites, apps, mobile cheque deposits, and third-party systems inaccessible to people with disability, as no alternative identity authentication methods are offered [21,25,28,29].

The accessibility of banking services is also compromised by the lack of assistive technology, e.g. induction loop systems at service counters, which can be a vital bridge to overcome disability-related hurdles. Moreover, existing banking software often exhibits incompatibility with screen readers and other assistive technology software [27,30]. In some cases, banking security software has been found to inadvertently block applications that implement assistive technology, rendering internet banking completely inaccessible [30].

In contrast, certain facilitators to inclusive banking have been identified and hold promise for enhancing digital financial inclusion. The implementation of accessibility features such as audio assistance, Artificial Intelligence (AI), and mobile applications (apps) that assist in identifying money has shown significant potential for people with a visual impairment [31]. Mobile money services, exemplified by platforms like M-Pesa, a simple mobile money platform developed in Kenya in 2007, have notably increased the number of women who can access formal financial services [32], and these have since expanded to other countries and regions. There is a growing demand for more inclusive features, such as online security devices with audio functions or ATMs that can be operated using speech and offer braille on the buttons [14,23].

The transition to more accessible electronic formats has also been met with a favourable response from people with disability. These formats, complemented by Braille, large print formats, and audio, can substantially improve the banking experience for people with disability. As the demand for inclusive features continues to rise, it is evident that the path towards truly inclusive banking involves tackling existing barriers while amplifying facilitators to enhance accessibility.

#### Services, systems and policies

In relation to services, systems, and policies, introducing digital finance in a country can result in exclusion unless banks ensure accessible information and preparation for all consumers, including people with disability [33]. Additionally, the lack of inclusion of people with disability (or voices from OPDs) in policies results in an absence of accommodations for their specific needs [30]. Various obstacles such as low financial literacy, the absence of AT, communication barriers, and high unemployment rates further contribute to commercial banks’ hesitancy to ensure financial inclusion for people with disability [30,31].

On the other hand, several facilitators were found to enhance financial inclusion. Government initiatives, exemplified by the Fijian government and the Malawi government’s National Strategy for Financial Inclusion, play a crucial role in promoting the financial inclusion of people with disability [15]. Adverts and outreach programs have also had a positive impact by encouraging the participation of people with disability in financial services, and raising awareness about available options [22]. Moreover, the implementation of accessibility policies and standards, particularly for websites, can be a significant step towards improving the accessibility of financial services for people with disability. However, adherence to these standards may vary [14].

In the absence of accessible mainstream financial services, people with disability are often left with limited options to manage their finances. This unfortunate circumstance renders them more susceptible to alternative financial services, which may not always have their best interests at heart. Predatory lenders, for instance, often take advantage of such situations, offering quick loans with excessively high-interest rates and unfavourable conditions. This exposes them to financial risks and additional hardships, potentially trapping them in cycles of debt and poverty. Hence, accessible financial services are crucial not just for financial inclusion but also to protect people with disability from exploitative financial practices, ensuring social justice [20].

#### Support and relationships

To navigate the barriers to accessing the financial sector independently, people with disability often rely on others to complete banking operations, such as sharing PIN codes or requiring assistance for visual authentication on websites [35]. This dependence on others can expose them to potential risks such as theft and fraud [21] and is in contravention of in-country data protection legislation.

Families’ involvement, on the other hand, can also improve access to banking services for people with disability, facilitating their financial inclusion [23,35,36]. In close-knit communities, the Community-Based Rehabilitation (CBR) training approach has shown broad benefits for both people with disability and non-disabled members, as demonstrated in Bangladesh [35]. Furthermore, other integrated robust support systems, such as designated support persons at ATMs, can enhance banking access for people with disability.

By recognising and addressing the barriers related to dependence and lack of support, while leveraging the facilitators of peer support, family networks, community support, and other strong support systems, efforts can be made to enhance financial inclusion for people with disability. Building inclusive support structures and promoting financial literacy within these networks can empower people with disability to access and navigate banking services more independently.

## Potential interventions

**Figure 5.**
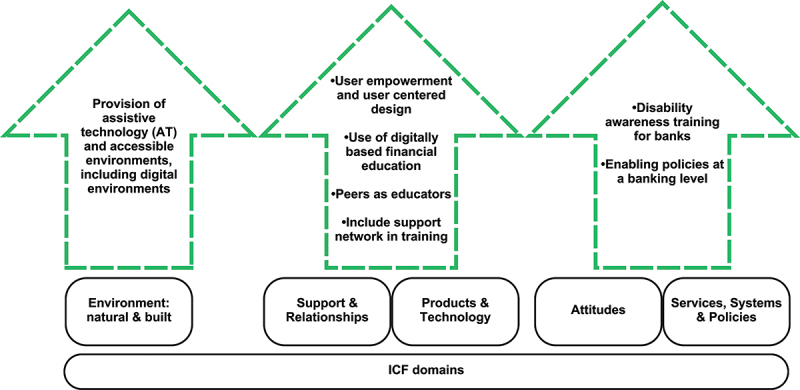
Potential interventions.

A number of potential interventions arise from the analysis (Figure 5) which are presented across the 5 ICF domains.

### Addressing people with disabilities lower demand for financial services

The Global Findex database (2021) suggests that people remain excluded from banking for different reasons, such as lack of money, lack of demand for formal financial services, distance from financial services providers, the high cost of maintaining a bank account, and lack of availability of appropriate financial services. However, the barriers restraining people with disability from entering the formal financial system are more nuanced and need more concerted efforts to overcome. For example, most people with disability have a lower demand for financial services [37]. Research suggests that such a lack of demand is primarily driven by cultural factors – e.g. the mindset that people with disability do not need to participate in the formal financial market [21]. Appropriately designed behaviour change communication modules directed at those without disability awareness can bring changes to behaviours, norms, and attitudes towards people with disability and their capabilities [38].

Furthermore, people with disability have low levels of financial literacy [39], which limits them from realising the importance of financial inclusion. Several financial literacy programs are designed for people with disability [40–43]. Interventions are required to evaluate the effectiveness of these programs on the financial capability of people with disability and their demand for banking services.

Finally, evidence suggests that people with disability are disproportionately represented among people experiencing poverty [44], and that poverty and unemployment partially explain why financially excluded individuals often decide not to open bank accounts [45]. Thus, strategies are required to enhance the employment opportunities and income-generating potential of people with disability. Interestingly, following the COVID-19 pandemic and the emergence of remote working accommodations, employment opportunities increased for people with disability [46]. Whilst not a solution for all, this suggests that increased employment inclusion is possible for some and may be feasible to implement.

### Use of digitally based financial education

ICT-based financial education programs can increase the level of financial literacy of people with disability and consequently help them overcome at least three of the five barriers mentioned above. It has been recognised in finance and economics literature that financial literacy training increases low-income persons’ demand for formal banking services through increasing financial literacy [37,47]. However, the effectiveness of such programs largely depends on the extent to which they can tailor to the needs of the target beneficiaries. The importance of these programs has also been recognised in disability policy literature, i.e. with improved financial knowledge, people with disability are less likely to be rejected for bank loans and other financial services [15]. An ICT-based financial education program can also support people with disability to overcome barriers to communication and web content accessibility.

In order to implement an ICT-based financial education program however it is necessary to address the gap in access people with disability face, to both digital devices like computers, and to address generally lower rates of access to the internet.

### Enabling policies for financial institutions

Many financially excluded individuals report that they do not go to banks because of geographical distance [45]. For people with disability this problem is accompanied by unfavourable physical environments and infrastructure, such as difficult terrains and inaccessible bank branches and ATMs [17]. Interventions are urgently required to improve the infrastructure and accessibility to banking services to accommodate the needs of people with disability. The literature shows that although accessibility standards exist within the financial sector, including for the physical environment, these are seldom implemented and do not take into account additional barriers that may be faced when trying to access financial services that may occur between someone’s home, and the bank [20]. A recent study reported that people with disability prefer cashless systems, particularly payment cards as opposed to complicated apps and trying to access inaccessible ATMs and banks [48].

### Provision of assistive technology and accessible environments, including digital environments

Existing evidence suggests that technology has played a vital role in improving financial inclusion over the last decade [49]. Assistive technology is also essential to facilitate greater financial accessibility [50]. Development of universally designed authentication methods and mobile banking mechanisms with features such as audio output is encouraged [28]. The role of ICT in these assistive technology solutions is implicit as these advancements rely on ICT for development and implementation. In the context of financially excluded people with disability, several studies underscore the significance of improved web accessibility in the banking sector, noting the current low compliance with existing guidelines, e.g. W3C [48,51]. One study mentioned that a very simple training program was successful in enabling participants with cognitive disabilities to withdraw money from an ATM, implying the efficacy of ICT-based training for enhancing financial literacy and interaction with banking services [52].

### User empowerment and user centered design

A user-centred design approach, including diverse user groups such as the elderly and people with disability in the development phase, is suggested as a model for success by several researchers including in South Africa [14]. Meaningful ideas were gathered from people with disability to increase access to financial services, for example, text messages as an alternative to call centres suggested by deaf customers, card-less ATM access suggested by those with mobility impairments, and increased audio inputs by those with vision impairment [14].

Implementing new access models would be supported by ICT-based training for staff and customers to ensure solutions are understood and used effectively.

## Discussion

This scoping review explores barriers and enablers of financial inclusion for people with disability in LMICs to inform appropriate interventions that may increase participation. The evidence gathered strongly indicates a pervasive issue of financial exclusion affecting people with disability worldwide. Despite the clear benefits of enhanced digital access to financial services, the digital divide combined with a lack of appropriate assistive technology leaves billions of unbanked globally [45]. Exclusionary practices appear to transcend the type of disability and intensify in relation to the severity of the disability [53]. Noting the low cost of implementing many of these simple interventions, it is clear that consultation with people with disability (and also OPDs) is crucial, and increasing inclusion in financial services is possible, as has already been proven in various countries and contexts.

Digital financial services have served as pivotal tools in promoting financial inclusion by extending user-friendly and secure banking services to people with disability. This not only enhances their individual overall spending capacity but, given that 16% of the world’s population is estimated to be living with a disability, has the potential to strengthen the GDP of developing economies through capitalising on the use of innovation and ICT [7,59]. Beyond economic benefits, accessible services offer increased financial control and independence to users, facilitating immediate financial decisions and transactions.

When considering the staggering rate of people with disability being unbanked [46], it is crucial to refocus this narrative through the perspective of disability justice. It is insufficient for financial services to merely exist – their design and application must take cognisance of and cater to accessibility needs. Financial policies and decisions that disregard people with disability not only overlook an opportunity for enhanced inclusion, but they also risk infringing upon broader human rights [3]. Thus, an inclusive approach is not simply optional, but an essential requirement in ensuring justice and equity in our increasingly digitised financial landscape.

It is also worth noting that although in some countries accessibility standards for the financial sector do exist, there is still a significant void in enforceable policy ensuring adherence to these standards by financial institutions. This ‘loophole’ marginalises those who are most in need of financial inclusion forcing them to look elsewhere for financial support, which includes predatory lending, and alternative financial ‘solutions’ that often result in poor outcomes, debt, and poverty.

## Recommendations

As detailed in Figure 5, our analysis distilled several dimensions into three primary potential intervention strategies to address financial exclusion among people with disability. These strategies focus on (1) countering attitudinal barriers within the financial sector, (2) proactively engaging in user-centred design to design more accessible banking and financial education, and (3) guaranteeing the accessibility of assistive technology along with accessible Information and Communication Technology (ICT) and other physical aspects of the banking sector.

The first intervention addresses the need for enhanced engagement by both people with disability and their representative groups (organisations of people with disability- OPDs) with financial systems, emphasising the role of ICT and education-based strategies. This approach also highlights the necessity of supportive policies to bridge the gap, where possible, between people with disability and financial services. The second intervention strategy focuses on societal attitudes towards people with disability. It examines the impact of these attitudes and the physical environment on the financial inclusion of people with disability. Lastly, the role of products and technology is at the forefront of the third intervention strategy. It stresses the importance of access, mobility, and ICT to facilitate interaction between people with disability and financial services. Despite the digital divide, unprecedented opportunities are available for the digital economy to support financial inclusion of people with disability. A more inclusive financial landscape can be achieved for people with disability through the digitalisation of financial intermediaries and financial products.

## Conclusion

Access to financial services is a human right that seems to have been overlooked for people with disability. In this rapidly evolving digital age, the push for financial inclusion of people with disability, particularly in LMICs, is not just a matter of economic strategy but a moral imperative rooted in justice and equity. The financial sector’s landscape, shaped by innovations in ICT, provides an unparalleled opportunity to bridge the financial divide faced by people with disability. However, mere technological advancements are not enough. True financial inclusion necessitates a paradigm shift in attitudes, policies, and strategies. Our findings underscore the urgency to redesign financial systems that are accessible to all and cognisant of the preferences and needs of people with disability.

Addressing the multiple dimensions of financial exclusion of people with disability requires a comprehensive, multifaceted approach, integrating attitudinal change, ICT accessibility, and a commitment to disability justice. As global partnerships like the Asian Development Bank, GDI Hub, and other stakeholders pave the way, there is an emphatic call for banks, policymakers, and society to converge their efforts.

## Data Availability

All articles included in this review are listed in Table 1 and are listed in the reference list. The complete set of data generated during this study are available from the corresponding author on reasonable request.
